# Expanding the toolbox: Emerging antisense oligonucleotide mechanisms for modulating gene expression

**DOI:** 10.1016/j.omtn.2026.102953

**Published:** 2026-05-14

**Authors:** Isabella Trew, Steve D. Wilton, Jessica M. Cale, May Aung-Htut

**Affiliations:** 1 Personalised Medicine Centre, Health Futures Institute, Murdoch University, Perth, WA 6150, Australia; 2 Perron Institute for Neurological and Translational Science, Perth, WA 6009, Australia

**Keywords:** MT: oligonucleotides: therapies and applications, antisense oligonucleotides, pre-mRNA splicing, polyadenylation, MicroRNA, miRNA, premature termination codons, upstream open reading frames, uORF, secondary structures, RNase H, gapmers, splice modulation

## Abstract

Decades of research have cemented antisense oligonucleotides (ASOs) as a cornerstone of molecular medicine. Advancements in synthesis and chemical modification, together with an improved understanding of the human genome and transcriptome, have enabled their emergence as highly specific and tailorable therapeutics across a wide variety of conditions. Although the greatest strength of ASOs lies in their nucleic acid composition that confers the theoretical ability to target any known genetic sequence, effective ASO design requires a holistic approach considering the molecular mechanism underlying the desired therapeutic outcome. Initially considered straightforward inhibitors of gene expression, ASOs have now evolved into versatile modulators capable of exploiting an increasingly diverse array of molecular processes. Despite this progress, their full therapeutic potential remains far from realized. Emerging research, driven by a deepening understanding of RNA biology, continues to expand the repertoire of mechanisms through which ASOs can modulate gene expression. Collectively, these studies demonstrate that ASOs can be designed to modulate numerous pre-mRNA processing events, including splicing, polyadenylation, microRNA activity, and translation initiation and termination, thereby broadening the range of conditions and patients that may benefit from ASO-based therapeutics.

## Graphical abstract

**Figure:**
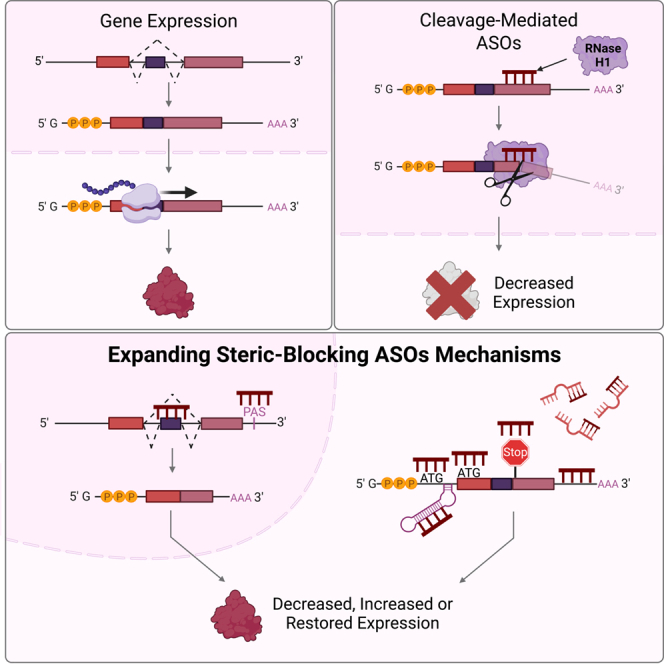

## Teaser

Emerging antisense oligonucleotide strategies extend beyond RNA degradation to modulate splicing, polyadenylation, and translation. This review examines the expanding mechanistic toolbox of ASOs and evaluates their translational potential, highlighting opportunities and limitations in extending their impact across diverse genetic and complex diseases.

## Introduction

Antisense oligonucleotides (ASOs) are short, synthetic nucleic acid analogs that have revolutionized the field of molecular medicine since their first report of suppressing viral replication in 1978.^1^ Typically comprising a single strand of 15–30 nucleotides, ASOs may anneal to complementary sequences through Watson-Crick base pairing and modulate target gene expression through a variety of mechanisms. The specific mechanism engaged is dependent on properties of both the target gene, such as transcript sequence, regulatory control, expression profile, and the desired direction of modulation, as well as of the ASO itself, such as chemical modifications and delivery efficiency to target cells. ASO design, therefore, necessitates a holistic approach in which chemistry and mechanism are carefully considered in the context of the pathological condition and target transcript.

As of February 2026, sixteen ASO drugs have been approved by the United States Food and Drug Administration (FDA) and European Medicines Agency (EMA) for use in patients diagnosed with a variety of conditions including Duchenne muscular dystrophy (DMD),^2^^,^^3^^,^^4^^,^^5^^,^^6^ spinal muscular atrophy (SMA),^7^^,^^8^^,^^9^ and amyotrophic lateral sclerosis (ALS).^10^^,^^11^ Modern ASOs often incorporate chemical modifications to the nucleotide backbone, sugar, and base moieties that are essential for enhancing nuclease resistance and on-target affinity, and reducing non-specific protein interactions, immune activation, and renal clearance.^12^^,^^13^^,^^14^^,^^15^^,^^16^^,^^17^ The most common chemistries seen in eleven FDA and EMA approved ASOs are the phosphorothioate (PS) backbone, which contains a sulfur atom in place of a non-bridging oxygen atom, in combination with the 2′-*O*-methoxyethyl (2′MOE) modification to 2′ position of the sugar moiety. Modifications to multiple moieties of the same nucleic acid may also occur, as demonstrated by four FDA approved drugs for the treatment of DMD, which are known as phosphorodiamidate morpholino oligomers (PMOs) and contain a morpholine ring and phosphorodiamidate linkages in place of the natural ribofuranose rings.^18^ Beyond modifications to the constituent nucleic acids of an ASO, enhancing delivery to target cells by conjugating molecules at the 5′ end is becoming more prominent. In fact, two recently approved PS-2′MOE ASOs are conjugated to a triantennary N-acetyl galactosamine (GalNAc_3_), a sugar that binds to the asialoglycoprotein receptor on the surface of hepatocytes to facilitate highly efficient cellular uptake and therefore enhances ASO potency in the liver.^19^^,^^20^ Although there are still several challenges to be overcome, such as the role of chemistry in toxicity (reviewed work by Bhamra et al.^21^) and inefficient delivery to many tissue types (reviewed work by Anand et al.^22^), it is evident that chemical modifications have allowed ASOs to advance significantly from their humble beginnings.

As our toolbox of chemical modifications has expanded, so too has the repertoire of mechanisms through which ASOs can modulate gene expression. Classically, ASOs have been used to simply reduce gene expression through engaging several endogenous degradation processes. Increasingly, however, they are designed to fine-tune various RNA processing events, including pre-mRNA splicing and polyadenylation, microRNA (miRNA) activity, and translation initiation and termination. The capacity to exert a diverse range of effects on their target is perhaps the greatest advantage of ASO therapeutics, and it is dictated by factors including chemical modifications, the specific region of ASO binding, type and function of target RNA, and the intended outcome. In fact, the field continues to rapidly evolve alongside advancements in technology and a deepening understanding of RNA biology, increasing the number of conditions amenable to ASO therapeutics and patients that may benefit. Although there may be exceptions, such as functional chimeras capable of producing multiple effects, ASO mechanisms typically fall into two broad categories according to whether they induce cleavage of target RNA, or sterically block RNA processing or translation. This review will examine the established molecular mechanisms underpinning the FDA and EMA approved ASOs to date, and explore emerging mechanisms through which ASOs can be designed to operate, highlighting the importance of aligning ASO design with both therapeutic context and molecular mechanism in mind.

## Cleavage-mediated ASOs

### RNase H1

**Figure 1:**
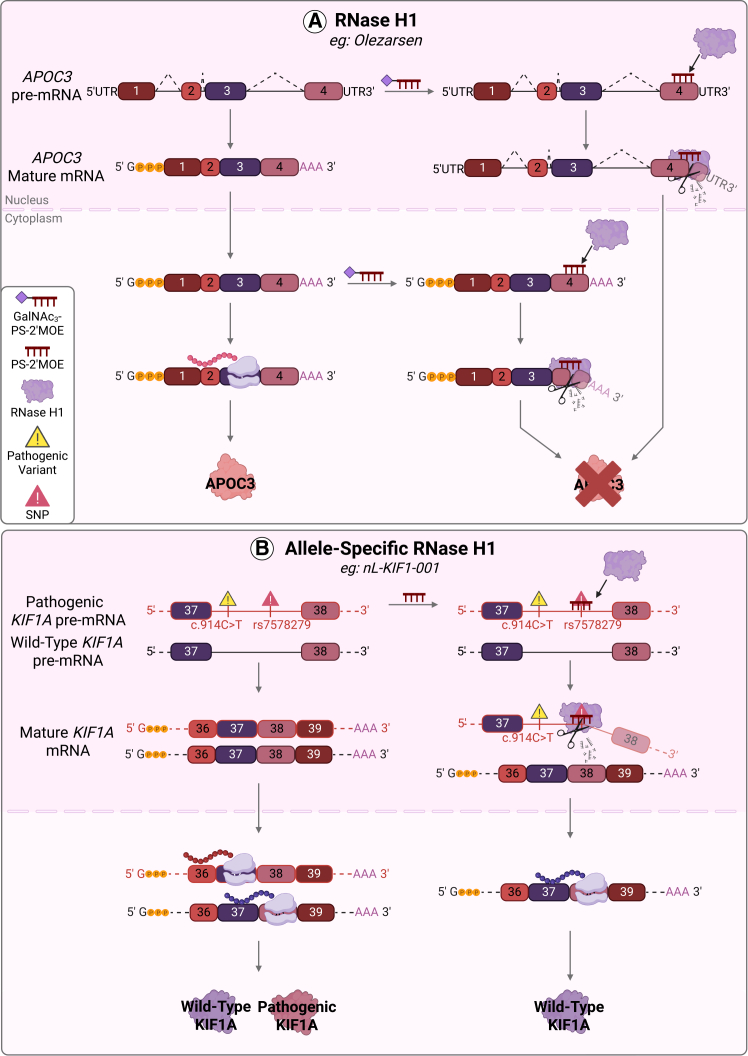
Recent RNase H1-mediated ASOs in the clinic and their mechanisms of action (A) Olezarsen is a PS-2′MOE conjugated to a triantennary N-acetyl galactosamine (GalNAc_3_) that anneals to *APOC3* pre-mRNA in the nucleus (top) or mature mRNA in the cytoplasm (bottom), triggering RNase H1 degradation to decrease APOC3 protein expression. (B) nL-KIF1-001 is a personalized PS-2′MOE that targets expression of the pathogenic allele encoding a dominant-negative protein by annealing to a SNP in intron 37 of pre-mRNA produced from the same allele. RNase H1 is activated and degrades pathogenic pre-mRNA, decreasing pathogenic KIF1A expression. Created in BioRender. Trew, I. (2026) https://BioRender.com/vs33f36. Schematic not drawn to scale.

ASOs can induce cleavage of target RNA sequences through several mechanisms, the most common and well-characterized of which are through the activation of the endogenous endonuclease RNase H1.^23^ Known as gapmers, these chimeric ASOs are designed such that a DNA core, which facilitates the formation of a DNA-RNA duplex with target mRNA through sequence complementarity, is flanked by “wings” of several 2′ modified nucleotides that increase oligomer stability and provide sufficient annealing specificity to the target transcript.^24^ The DNA-RNA duplex is then recognized and the RNA strand is degraded by RNase H1, subsequently suppressing protein production.^25^ There are nine ASOs approved by the FDA and EMA that utilize this mechanism, including olezarsen, which received approval in 2024 to treat severe hypertriglyceridemia in patients diagnosed with familial chylomicronemia syndrome (FCS).^26^^,^^27^ Olezarsen is also one of the two approved ASO drugs conjugated to a GalNAc_3_ molecule that facilitates efficient uptake into hepatocytes, where it anneals to apolipoprotein C3 (*APOC3*) mRNA that encodes a protein component of triglyceride-rich lipoproteins (Figure 1A). The DNA-RNA duplex formed then activates RNase H1, resulting in the cleavage of *APOC3* mRNA and decreased production of APOC3, thereby lowering circulating triglyceride levels.^26^^,^^27^ Other approved ASO drugs, including their date of approval/discontinue, chemistry, gene or disease target, and mechanism of action, are outlined in Table 1.

**Table 1:** Characteristics of current and historic FDA and EMA approved antisense oligonucleotide drugs

Drug	FDA approval/discontinue	EMA approval/discontinue	Chemistry	Pathology	Mechanism
**Active**					
Eteplirsen	2016	–	PMO	DMD	splice modulation: *DMD* exon 51 skipping^2^^,^^6^
Nusinersen	2016	2017	PS-2′MOE	SMA	splice modulation: inclusion of *SMN2* exon 7^7^^,^^8^^,^^9^
Inotersen	2018	2018	PS-2′MOE	ATTR	RNase H1-mediated: degradation of *TTR* mRNA^28^^,^^29^
Golodirsen	2019	–	PMO	DMD	splice modulation: *DMD* exon 53 skipping^3^
Volanesorsen	–	2019	PS-2′MOE	FCS	RNase H1 mediated – degradation of *APOC3* mRNA^30^^,^^31^
Viltolarsen	2020	–	PMO	DMD	splice modulation: *DMD* exon 53 skipping^4^
Casimersen	2021	–	PMO	DMD	splice modulation: *DMD* exon 45 skipping^5^
Tofersen	2023	–	PS-2′MOE	ALS	RNase H1-mediated: degradation of *SOD1* mRNA^10^^,^^11^
Eplontersen	2023	–	GalNAc_3_-PS-2′MOE	ATTR	RNase H1-mediated: degradation of *TTR* mRNA^32^
Olezarsen	2024	–	GalNAc_3_-PS-2′MOE	FCS	RNase H1-mediated: degradation of *APOC3* mRNA^26^^,^^27^

**Individualized/compassionate use**					

Milasen	2018	–	PS-2′MOE	Batten disease	splice modulation: correction of *MFSD8* intron 6 cryptic splice site^33^
Valeriasen	2020	–	PS-2′MOE	DEEs	RNase H1-mediated: degradation of *KCNT1* mRNA^34^
Atipeksen	2020	–	PS-2′MOE	A-T	splice modulation: correction of *ATM* exon 53 cryptic splice site^35^
nL-KIF1-001	2024	–	PS-2′MOE	KAND	RNase H1-mediated: degradation of pathogenic allele-specific *KIF1A* mRNA^36^

**Discontinued**					

Fomivirsen	1998 (2006)	1999 (2002)	PS	CMV retinitis	RNase H1-mediated: degradation of *IE2* mRNA^37^
Mipomersen	2013 (2019)	–	PS-2′MOE	HoFH	RNase H1-mediated: degradation of *ApoB-100* mRNA^38^^,^^39^

FDA, Food and Drug Administration; EMA, European Medicines Agency; PS, phosphorothioate modified backbone; 2′MOE, 2′-*O*-methoxyethyl; PMO, phosphorodiamidate morpholino oligomers; GalNAc_3_. triantennary N-acetyl galactosamine; DMD, Duchenne muscular dystrophy; SMA, spinal muscular atrophy; ATTR, transthyretin amyloidosis; FCS, familial chylomicronemia syndrome; ALS, amyotrophic lateral sclerosis; DEEs, developmental and epileptic encephalopathies; A-T, ataxia-telangiectasia; KAND, KIF1A-associated neurological disorder; CMV, cytomegalovirus; and HoFH, homozygous familial hypercholesterolemia.

Another recent example is nL-KIF1-001, which was granted approval in 2024 on compassionate grounds to treat a patient living with KIF1A-associated neurological disorder caused by a heterozygous *de novo* pathogenic variant in one copy of the *KIF1A* gene (Table 1).^36^ In this case, nL-KIF1-001 anneals to a heterozygous single nucleotide polymorphism (SNP) present in *KIF1A* transcripts produced from the pathogenic allele and triggers RNase H1 activation, thereby degrading pathogenic transcripts (Figure 1B). Although follow-up is limited to 9 months, moderate improvement was reported across several symptoms. Together with the occurrence of only a single serious adverse event attributed to complications of the first intrathecal injection, this study supported the feasibility of allele-specific ASOs for therapeutic benefit.^36^ Allele-specific gapmers have also been evaluated in phase Ib/IIa clinical trials for the treatment of Huntington’s disease. Similar to nL-KIF1-001, these ASOs target a heterozygous SNP present in the huntingtin (*HTT)* allele harboring the causative autosomal dominant expansion repeat, thereby causing degradation of pathogenic transcripts.^40^ Developed by Wave Life Sciences Ltd., trials of gapmer ASO drug WVE-003 indicate a promising reduction of pathogenic HTT expression, while critically maintaining expression of wild-type HTT (NCT05032196).^41^ Development of genetic therapeutics for conditions driven by heterozygous gain-of-function variants in critical genes has long proven difficult, as most approaches involve the knockdown of both pathogenic and wild-type alleles, thereby causing further deleterious effects. The capacity of gapmer ASOs to act in an allele-specific manner for such conditions exemplifies their power and precision, with pre-clinical investigation ongoing for the treatment of conditions such as spinocerebellar ataxia type 3.^42^

Since it is reported that gapmers activate and recruit RNase H1 to cleave target RNA within both the cytoplasm and the nucleus,^43^ mature transcripts in addition to nuclear precursors, including precursor ribosomal RNA (pre-rRNA) and pre-mRNA, may also be targets for ASO design. Furthermore, non-coding RNAs, including long non-coding RNAs (lncRNAs)^44^ such as natural antisense transcripts (NATs),^45^^,^^46^ promoter-associated RNAs,^47^ as well as circular RNAs^48^ are emerging as potential ASO targets. Notably, targeting these nuclear non-coding RNAs allows ASOs to modulate gene expression not only at the post-transcriptional level but also through transcriptional and chromatin-associated mechanisms. Indeed, gapmers targeting these nuclear RNAs are proving to be a promising approach in pre-clinical studies for anti-cancer drug design, including for multiple myeloma^44^ and juvenile myelomonocytic leukemia,^49^ and are beginning to reach clinical trials for glioblastoma (jRCT2041230136).^50^ The most prominent example of ASO-mediated modulation of a non-coding RNA is rugonersen, a gapmer targeting the paternal NAT, referred to as *UBE3A-ATS*, which mediates neuron-specific imprinting of the ubiquitin-protein ligase E3A (*UBE3A*) gene by transcriptionally silencing the paternal allele.^51^ This approach is under investigation as a therapeutic strategy for Angelman syndrome, whereby the induction of RNase H1-mediated degradation of *UBE3A-ATS* relieves silencing of the paternal allele, with the aim of compensating for the loss of the maternal allele.^45^^,^^52^ In 2025, a phase I clinical trial of rugonersen (NCT04428281) reported an acceptable safety and tolerability profile, along with promising preliminary efficacy indicators including improvements in abnormal brain activity and developmental measures in children aged 1–12 years carrying pathogenic *UBE3A* variants.^46^ Rugonersen provides proof-of-concept for the use of gapmer ASOs to reactivate silenced imprinted genes and, although yet to be investigated further, could represent a viable therapeutic approach for other lncRNAs implicated in imprinting disorders.

While gapmers have greater flexibility in terms of target sequence, as they typically are not required to anneal a particular motif or site, such as the targeting of splicing consensus sequences in splice modulating ASOs (discussed in following sections), the predominant concern for their design is the propensity for off-target effects driven by their sequence, even in cases of partial complementarity. Indeed, mismatches or full complementary in as little as 7–10 continuous bases have been demonstrated to activate RNase H1 and trigger cleavage of unintended RNAs.^53^^,^^54^ Sequence extension or the incorporation of different chemical modifications may reduce off-target effects;^55^ however, these should be chosen carefully to avoid inducing chemistry-driven off-target effects, a key consideration for all ASO designs (reviewed work by Crooke et al.^56^). Therefore, while gapmers have proved their potency and therapeutic value in contexts where complete knockdown of an RNA target, including those produced from a specific allele, is desirable, their tendency to cause off-target effects should be carefully considered during design and approval.

### AGO2 and RNase L

Beyond RNase H1 activation, other cleavage-mediated ASO mechanisms have been investigated. The most well-known of these is the design of functional chimeric ASOs that act as substrates for argonaute 2 (AGO2), a key catalytic component of the RNA-induced silencing complex (RISC) involved in the RNA interference pathway. Although difficult due to structural requirements, ASOs may be designed to act as substrates for AGO2, which activates RISC to catalyze the cleavage or inhibit translation of target mRNA, effectively silencing gene expression.^57^ Specifically, a 2012 study by Lima et al. reported heavily modified single-stranded siRNAs that were active *in vivo*. These siRNAs, which are in essence ASOs, exhibited AGO2-mediated knockdown of phosphatase and tensin homolog deleted on chromosome 10 (*PTEN*) mRNAs.^58^ However, this chimera ASO was heavily modified, containing a phosphate analog at 5ʹ end to enhance stability and binding to AGO2, alternating 2ʹfluoro and 2ʹ-*O*-methyl (2ʹOMe) nucleosides, and a modified 2ʹOMe adenosine dinucleotide at the 3ʹ end for interactions with the Dicer ribonuclease involved in the RISC assembly. Without these heavy modifications and stringent sequence requirements, the ASOs were not active *in vivo*.^58^ These stringent requirements for chemical and sequence composition plausibly limit the applicability of this ASO mechanism to only a small number of target genes. Indeed, there have been no further reports of ASOs that engage this pathway, including to targets beyond *PTEN* mRNA, to the authors’ knowledge.

Another example of cleavage by a functional chimeric ASO includes those that engage in the RNase L pathway, an innate immune response that functions to degrade viral RNA upon infection. This pathway involves the production of 2′ to 5′ linked oligoadenylates (2′-5′A), small oligonucleotides comprising a single-stranded sequence of adenyl nucleosides linked by 2′ to 5′ phosphodiester bonds that bind to and activate RNase L.^59^ In this case, the cytoplasmic nuclease, as opposed to the nuclear and cytoplasmic RNase H1, then cleaves nearby viral and host RNA.^60^ When conjugated to an ASO that anneals to a complementary target sequence, 2′-5′A elements may activate RNase L and trigger cleavage of nearby host RNA, theoretically including the target RNA.^61^ Most reports suggest that 2′-5′A-linked ASOs leverage the immune response involvement of RNase L to function as an antiviral drug, such as a recent report of a chimera comprising a 2′-OMe-modified ASO linked to a 5′-phosphorylated 2′-5′A that was capable of targeting viral RNAs encoding the spike and envelope proteins in SARS-CoV-2 infection.^62^ While this mechanism may be desirable in the context of antiviral development, the natural involvement of RNase L in an inflammatory response and its participation in feedback loops that promote an antiviral response would likely be problematic in other pathologies, particularly in those already exacerbated by inflammation.^63^ In addition to the cleavage of viral or host transcripts, RNase L is reported to result in other significant changes to the host transcriptome, such as altering translation termination and the consequent expression of host mRNAs, including those not involved in the antiviral response.^64^^,^^65^^,^^66^ Critical cellular processes of the host, such as differentiation, proliferation, senescence, apoptosis, and tumorigenesis, are also impacted by RNase L activation.^65^^,^^67^ Furthermore, additional research is required to determine if the high specificity carried by the ASO element of the chimera is sufficient to overcome the inherent lack of cleavage specificity by the RNase L system, as studies thus far have focused predominantly on the on-target effects. Therefore, 2′-5′A-linked ASOs remain in pre-clinical development.

**Figure 2:**
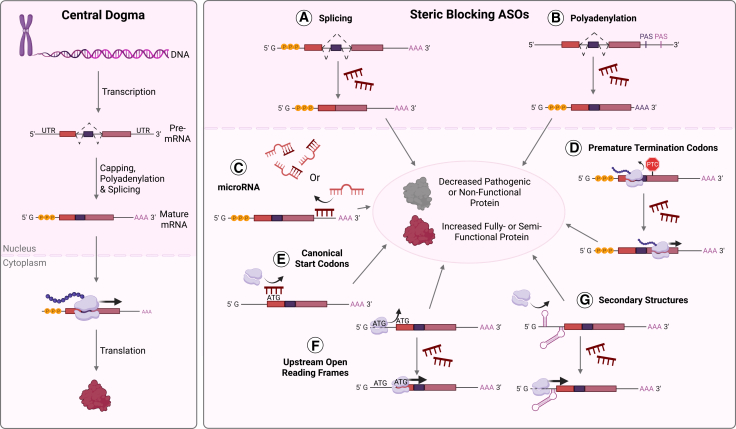
Steric-blocking ASO mechanisms of action ASOs can be designed to alter target protein expression by modulating steps within the central dogma (left) including pre-mRNA processing events (A and B), post-transcriptional regulation mediated by microRNAs (C), and translational control through modulation of termination and initiation (D–G). Depending on the target gene and the desired direction of modulation, these approaches may reduce the production of pathogenic or non-functional proteins, or restore or enhance the production of fully or partially functional proteins. Created in BioRender. Trew, I. (2026) https://BioRender.com/j63gp8w. Schematic not drawn to scale.

In addition to causing the cleavage of target RNA, ASOs may also alter protein expression or structure through a variety of mechanisms that interfere with pre-mRNA processing events, such as splicing or polyadenylation, post-transcriptional regulation by miRNAs, and translation initiation and termination. Referred to as steric-blocking due to their nature of interfering with or “blocking” processing or translation events, the diverse repertoire of mechanisms in our toolbox is ever-expanding and allows incredibly precise modifications attuned to a specific disease context (Figure 2). Henceforth, this review will focus on the established and newly reported steric-blocking approaches of utilizing ASOs.

### Steric-blocking ASOs

#### Pre-mRNA splicing

**Figure 3:**
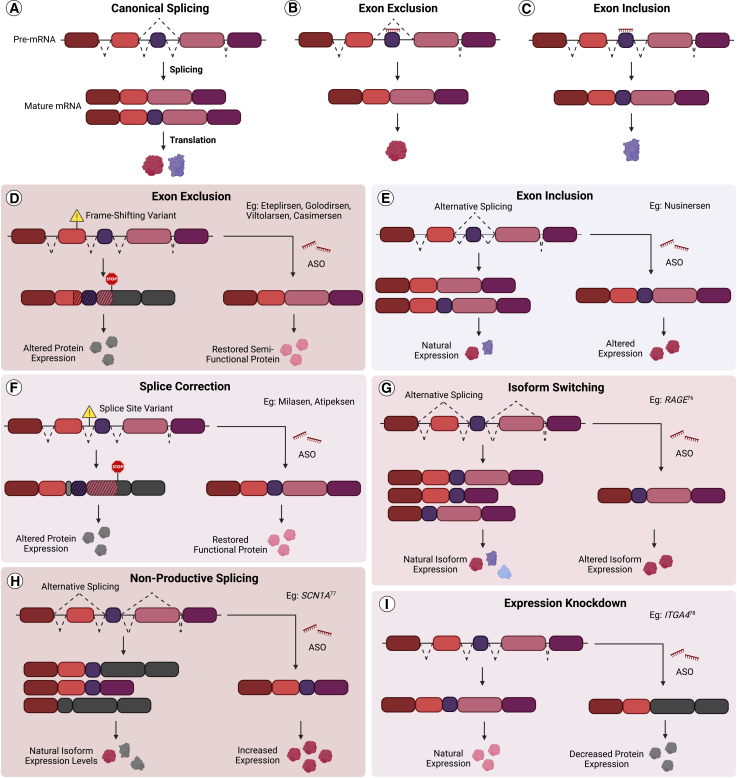
Pre-mRNA splicing and approaches for ASO-mediated modulation of splicing (A) Canonical splicing in RNA processing involves the pre-mRNA removal of non-coding introns and precise ligation of coding exons to create mature mRNA transcripts for translation. ASOs can be designed to alter splicing by promoting (B) exon exclusion or (C) exon inclusion for therapeutic benefit. (D–F) Examples of FDA approved ASO drugs that modulate splicing by (D) excluding exons, (E) including exons, (F) correcting splicing variants and modulating splicing to (G) switch isoforms, (H) increase isoform expression, or (I) decrease isoform expression. Created in BioRender. Trew, I. (2026) https://BioRender.com/th8coxw. Schematic not drawn to scale.

Modulating pre-mRNA splicing is the most established and successful mechanism of steric-blocking ASOs, with seven of the FDA approved drugs operating by this mechanism (Table 1). Splicing occurs during pre-mRNA processing and involves the removal of non-coding introns and precise ligation of coding exons together to create the mature transcript for translation (Figure 2A). Exons are often removed or included under various circumstances during natural alternative splicing, a process that synthesizes multiple transcripts from a single gene (Figure 3A).^68^ Splicing is directed by the presence of numerous motifs that define sequences as exonic or intronic and are recognized by the splicing machinery in the exon definition model. These sites include 5′ donor and 3′ acceptor splice sites, which designate exon/intron boundaries, as well as polypyrimidine tracts, branchpoints, and splicing enhancer and silencer-binding sites, which increase or decrease definition strength.^69^^,^^70^ These elements are exemplary targets for ASO design in a variety of pathological contexts whereby the exclusion or inclusion of exons or introns, as well as correction of splicing abnormalities (Figures 3B and 3C), may be applied to restore fully- or semi-functional protein expression.

As of February 2026, a total of seven splice modulating ASOs have received FDA and EMA approvals for the treatment of patients diagnosed with various conditions. The first was eteplirsen,^2^^,^^6^ a PMO pioneered by our laboratory^71^^,^^72^ that was approved by the FDA in 2016 for a subset of patients diagnosed with DMD, followed by golodirsen,^3^ viltolarsen,^4^ and casimersen^5^ in 2019, 2020, and 2021, respectively. These drugs are designed to treat a subset of patients carrying homozygous exonic deletions within the dystrophin (*DMD)* gene that result in a frameshift and creation of premature termination codons (PTCs); therefore, *DMD* pre-mRNAs harboring these variants are subject to degradation by the endogenous nonsense-mediated decay (NMD) pathway.^73^ By promoting the exclusion or “skipping” of exons adjacent to the sites of commonly reported variants, these drugs modulate pre-mRNA splicing to restore the reading frame of mature mRNA and allow translation of an internally truncated DMD protein, an example of two wrongs making a right (Figure 3D).^6^^,^^71^^,^^72^

Another prominent splice modulating ASO is nusinersen, an FDA and EMA approved drug for the treatment of SMA (Table 1). Nusinersen operates by leveraging the almost identical survival of motor neuron 2 (*SMN2)* gene to compensate for the loss of the critical SMN protein typically caused by whole gene deletions of *SMN1*. By targeting the intronic splicing enhancer (ISS-N1) motif downstream of *SMN2* exon 7, nusinersen promotes the inclusion of exon 7 in the mature mRNA and, therefore, allows the production of fully functional SMN protein.^74^^,^^75^ As such, nusinersen is typically described to operate by promoting exon inclusion (Figure 3E). However, it may also be considered a nuanced example of isoform switching, since natural alternative splicing of *SMN2* produces low levels of transcripts containing exon 7 compared to the pre-dominant exclusion of exon 7. Isoform switching ASOs are used to specifically alter inclusion or exclusion of exons or introns to change isoforms in alternatively spliced genes. In this case, modulation of *SMN2* exon 7 splicing switches the production of transcripts encoding an unstable, truncated protein isoform to the full-length SMN protein. This mechanism is undergoing further pre-clinical exploration in other contexts, such as the conversion of pro-inflammatory to anti-inflammatory isoforms of receptor for advanced glycation endproducts (*RAGE*) by altering the splicing of exons encoding for transmembrane domains^76^ (Figure 3G).

In addition to switching between protein-coding transcripts, the modulation of non-coding transcripts is also undergoing pre-clinical evaluation. A 2020 study by Lim et al. reported naturally existing transcripts that are subject to NMD, and are therefore, non-coding, may be switched to protein-coding full-length transcripts by ASO treatment, subsequently increasing the expression of mRNA and protein.^77^ This study utilized ASOs to alter splicing of non-coding transcripts produced from four target genes, one of which was the sodium voltage-gated channel alpha subunit 1 (*SCN1A*) gene, which showed increased protein-coding transcript expression *in vitro* as well as *in vivo* protein levels following ASO treatment (Figure 3H).^77^ Autosomal dominant loss-of-function variants in *SCN1A* lead to haploinsufficiency of the SCN1A voltage-gated sodium channel essential for neuronal activity that underlies Dravet syndrome. This approach is termed targeted augmentation of nuclear gene output (TANGO), and it is the proprietary platform of Stoke Therapeutics that provides remarkable proof-of-concept that such ASOs may be a valid therapeutic strategy for other autosomal dominant haploinsufficiency conditions or any condition that may benefit from the upregulation of suitable genes through suppression of splicing in non-coding transcripts. Furthermore, the opposite may also be true whereby protein-coding mRNA and protein expression may be reduced through the alteration of splicing and induction of NMD (Figure 3I).^78^ In this case, ASOs are designed to remove out-of-frame exons from wild-type protein-coding transcripts, subsequently altering the reading-frame of mature mRNA and creating PTCs which are degraded. This approach may be suitable for any condition that may benefit from the downregulation of suitable genes, such as many cancer or inflammation contexts. Although ASOs utilizing these mechanisms are yet to reach clinical trials, these reports demonstrate the flexibility of ASO-mediated splice modulation as a therapeutic approach and further increases the conditions potentially amenable to their intervention.

As mentioned, ASOs are proving powerful in the field of personalized medicine to treat a range of conditions, particularly those driven by pre-mRNA splicing abnormalities. Variants may disrupt canonical splice sites and create or activate alternative donor or acceptor sites, known as cryptic splice sites, often leading to the production of aberrantly spliced transcripts that can be pathological, depending on the affected gene and type of variant. While disruptions to the canonical splice site itself are considered difficult to correct, variants located elsewhere that disrupt canonical splice site recognition, or create or activate cryptic splice sites, may be promising targets for ASO design (Figure 3F). ASOs have proven powerful in correcting these splicing events and restoring partial or full protein functionality, the first of which was developed by Kim et al. in 2019.^33^ Known as milasen, this variant-specific ASO was designed to block the cryptic splice acceptor site i6.SA and adjoining splice enhancer in the major facilitator superfamily domain containing 8 (*MFSD8*) gene, which was activated by a SINE-VNTR-Alu (SVA) insertion, in a patient diagnosed with a rare form of Batten disease. This 22mer PS-2′MOE modified ASO corrected the splicing abnormality, improved wild-type MFSD8 expression, and received remarkably accelerated approval for compassionate use, with patient administration occurring within one year of variant identification. This precedent was followed in 2020 by the approval of another variant-specific ASO known as atipeksen for treatment of a patient diagnosed with ataxia-telangiectasia (A-T).^35^ Development of ASOs that correct similar aberrant splicing continues for variants associated with conditions such as Fukuyama muscular dystrophy.^79^^,^^80^

In contrast to cleavage-mediated mechanisms, steric-blocking ASOs are considered less likely to induce direct transcript degradation due to the lack of endonuclease-activating domains; however, they may still exert off-target effects through unintended modulation of splicing, translation, or RNA-protein interactions at partially complementary sites.^81^ In contrast to the link between chemical modifications and off-target effects, the capacity of steric-blocking ASOs to cause off-target effects is not well-characterized as most investigations do not take mechanism into account, thus limiting direct comparisons. Furthermore, while splice modulation is the most successful steric-blocking ASO mechanism to date and is particularly useful for the correction of splicing variants, it is not an appropriate therapeutic approach for many other disease contexts and genes. For example, splice modulation would not be suitable for variants located within critical functional domains wherein exon removal would be further deleterious. This is predicted to be the case for cystic fibrosis as missense variants and therefore, single amino acid changes have been reported to disrupt *CFTR*^82^ and confer symptoms in patients; therefore, loss of the entire exon harboring such a variant is expected to be catastrophic. As such, it is critical to develop and fill our toolbox with other approaches.

#### Polyadenylation

Beyond pre-mRNA splicing, ASOs may also be designed to modulate other crucial RNA processing steps including 5′ cap formation and 3′ cleavage and polyadenylation. Several RNA-binding proteins and sequence motifs are essential to initiate and perform these modifications, which generate mature transcripts that are stable against exonucleases, can be exported from the nucleus, and promote translation. Steric blockade of these elements by ASOs has been reported to alter the generation and stability of mature mRNA in various contexts. For example, ASOs have been utilized to interfere with polyadenylation sites (PASs) in the 3′ untranslated region (UTR) of mRNA, the non-coding sequence typically located immediately downstream of the coding region, and achieve knockdown of target mRNA and protein expression, with potential applications in gain-of-function pathologies (Figure 2B).^83^

As a result of natural alternative splicing, isoforms may also possess differing 3′ UTRs with alternative PASs. In addition to modulating the exon composition of 3′ UTRs, ASOs have also been investigated to block PAS usage, therefore redirecting polyadenylation to another PAS associated with a different isoform.^84^^,^^85^ By suppressing polyadenylation, targeted isoforms possess impaired nuclear export and shorter half-life, decreasing mature mRNA and therefore, protein expression of that isoform, and in turn promoting the polyadenylation of other isoforms, thereby increasing their total or fractional mRNA and protein expression.^84^^,^^85^ Similarly, PASs may also be present within the exons or introns of the coding sequence of transcripts that encode for a truncated protein, or are non-protein coding. Steric blockade of these sites using ASOs also allows isoform switching^85^ and represents a potential therapeutic strategy in contexts such as allergies^85^ and cancer.^86^ Furthermore, as many lncRNAs are subject to similar RNA processing including splicing, polyadenylation, and 5′ capping, these are also potential targets for ASO-mediated modulation. For example, ASOs that suppress an internal PAS to switch expression between two major isoforms of the nuclear paraspeckle assembly transcript 1 (*NEAT1*) lncRNA have been investigated as an anti-cancer approach.^86^ In this case, blockade of an internal PAS redirects polyadenylation from the growth-promoting *NEAT1* isoform, thereby decreasing protein expression, to the growth-inhibiting isoform, subsequently increasing protein expression. Recent studies also suggest the potential for ASOs to modulate cryptic PASs. These non-canonical sites may be created or activated in some pathological contexts to produce truncated proteins. However, evidence of directly modulating the PAS itself as opposed to suppressing the splicing of a cryptic exon containing a PAS, such as the case in stathmin-2 (*STMN2*) in ALS,^87^ is yet to emerge. Nevertheless, this approach aligns with established reports demonstrating the ability to suppress PASs and the effect of removing cryptic PASs indirectly, indicating plausibility.

Although no polyadenylation modulating ASOs have entered clinical trials to date, this mechanistic addition to our toolbox may increase the number of conditions potentially amenable to ASO therapies. However, the mechanisms by which ASOs interfere with polyadenylation remain poorly understood, such as whether they act to suppress cleavage or elongation of the poly(A) tail. Furthermore, accurate characterization and sequencing of 3′ UTRs is essential for the design of these ASOs, and although we can now map novel isoforms by long-read sequencing, our knowledge of their expression across cell type, developmental stage, and stress is still limited.

Reports of steric blockade of 5′ cap formation are considerably less common. In fact, while ASOs can target the 5′ UTR, the non-coding sequence typically located immediately upstream of the coding region, for translation inhibition (discussed in the following sections), this has been linked to modulating translation inhibitory elements (TIEs) or suppression of translation initiation, with no specific reports of specifically inhibiting cap formation. This is perhaps due to the almost immediate nature in which 5′ capping occurs following transcription initiation,^88^ therefore limiting the time available for ASOs to anneal to the mRNA and prevent addition of the 7-methylguanosine base. As such, inhibition of capping by ASOs remains predominantly theoretical, and further work is required to validate this mechanism as a potential ASO approach.

#### miRNA activity

ASOs may be designed to target miRNAs and suppress their activity either by complementary interactions with the miRNA molecules themselves, resulting in their sequestration or degradation, or masking their binding sites within the 3′ UTR (Figure 2C). AntimiRs, also referred to as antagomiRs, are ASOs that bind directly to mature miRNAs and may be either gapmer or steric-blocking ASOs. An example is miravirsen, the first antimiR to enter clinical trial, which targets the liver-specific miR-122 that promotes hepatitis C viral replication by stabilizing viral RNA.^89^ Miravirsen operates by binding and forming a stable heteroduplex with miR-122, thereby sequestering mature miR-122 and preventing its interaction with viral RNA as a treatment for chronic hepatitis C viral infections (NCT01200420).^89^ Although there are currently no antimiR drugs approved by the FDA or EMA, several compounds are under evaluation in ongoing clinical trials, predominantly as treatments for various cancers due to mounting evidence surrounding the role of miRNAs in the tumor microenvironment.^90^ While antimiRs have yet to achieve success in clinical trials for the treatment of rare genetic conditions, pre-clinical development continues for conditions such as myotonic dystrophy, where improved muscle function has been reported in mouse models of myotonic dystrophy type 1 following treatment with antimiRs targeting miR-23 b and miR-218.^91^^,^^92^ These miRNAs are natural translational repressors of the alternative splicing regulatory proteins MBNL1/2 that are sequestered and depleted by pathogenic *DMPK* transcripts containing CUG expansions in myotonic dystrophy. By annealing to and inhibiting the role of these suppressors, MBNL expression and downstream dysregulated genes are recovered in patient-derived myoblasts.^92^

A major challenge to the feasibility of antimiRs is the sheer complexity of miRNA expression and their plethora of gene targets. With more than 2,000 different miRNAs characterized in human cells and constant expansion of databases through multi-omics technologies, these molecules exhibit dynamic temporal and spatial expression patterns.^93^ In fact, single miRNA molecules are known to regulate multiple pathways through their interactions with numerous target genes, rendering them both a complex and potentially powerful ASO target,^94^ depending on the target miRNA. In contrast to targeting the miRNA itself, designing ASOs that sterically block the complementary binding sites in the 3′ UTR of a specific target RNA could mitigate this potential for global effects and increase safety. For example, a 2023 study reported increased progranulin (GRN) protein expression following treatment with ASOs that anneal to and sterically block the binding site of miR-29 b in the 3′ UTR of *GRN* mRNA.^95^ Heterozygous loss-of-function variants in *GRN* result in haploinsufficiency of the glycoprotein that is critical for lysosomal homeostasis in neurons and microglia, leading to the development of neurodegenerative conditions such as frontotemporal dementia. By blocking the binding site of the negative regulatory miR-29 b using an ASO, this study reported increased GRN expression in iPSC-derived cortical neurons and a humanized *GRN* mouse model.^95^ Although yet to be validated in a background harboring GRN haploinsufficiency, this study demonstrates the potential to target miRNA activity in a manner specific to a single target gene, and perhaps its isoforms. While this approach may reduce the risk of broader transcriptome-wide effects associated with direct miRNA targeting, it relies on accurate characterization and sequencing of 3′ UTRs, many of which vary extensively due to alternative polyadenylation that is highly tissue- and context-dependent, and remains incompletely resolved by current long-read sequencing and annotation strategies. Nevertheless, this approach may become a viable addition to our toolbox as our knowledge of miRNA function and mapping of 3′ UTRs continues to expand.

#### Premature termination codons

As discussed, the creation of PTCs by nonsense variants, or a frameshift that brings a stop codon into frame, can trigger NMD or lead to the production of truncated proteins that are partially functional, non-functional, or even toxic. Such variants are the driving factor for many pathologies including DMD, cystic fibrosis, and Rett syndrome. However, not all variants are candidates for reading frame correction by splice modulating ASOs, and other mechanisms to address these conditions are required.

ASOs that modulate the NMD pathway to reduce degradation of PTC containing transcripts in general, thereby promoting their translation and acting as a readthrough agent, have been investigated. For example, decreasing the expression of NMD factors SMG1 and SMG6 has been reported to increase translation of functional cystic fibrosis transmembrane conductance regulator (CFTR) as a therapeutic avenue for cystic fibrosis.^96^^,^^97^ However, like other readthrough agents, this approach is non-specific as it targets the NMD pathway itself and may lead to global effects on the transcriptome, such as miscoding or readthrough of canonical stop codons and aberrant protein production.^98^^,^^99^

To mitigate this, a recent study by Susorov et al. utilized ASOs to direct specific readthrough in combination with readthrough agents.^100^ In this approach, readthrough ASOs (R-ASOs) were designed to anneal downstream of PTCs where they modulate translation termination by interfering with target mRNA ribosomal entry and stop codon recognition (Figure 2D). In the presence of non-specific readthrough agents that promote elongation of mRNAs such as aminoglycosides and suppressor tRNAs, R-ASOs are reported to increase PTC-containing protein production without interfering with the expression of the wild-type gene.^100^ Additionally, this study demonstrated that R-ASOs tolerate chemical modifications to a certain degree, and their efficiency is highly dependent on the sequence surrounding the targeted stop codon and therefore, stop codon strength, suggesting that a degree of specificity is conferred by the ASO.^100^ Consequently, R-ASOs may prove useful in not only improving the specificity and safety of readthrough agents, but they also represent a novel ASO mechanism for conditions caused by nonsense variants that are not candidates for splice modulating ASO design. Such variants may include those that create a PTC within or adjacent to exons critical for protein function, or occur in the absence of reading frame disruption. However, it is important to note this *in vitro* work is preliminary and performed using nanoluciferase reporter assays in cell lysate.^100^ Crucially, functionality of the resultant proteins produced from the readthrough is yet to be confirmed, and off-target effects of the readthrough agent is yet to be reported. Nevertheless, this represents an exciting new approach for the design of ASOs capable of modulating nonsense variants present in numerous pathologies and a promising addition to our therapeutic toolbox.

#### Canonical start codons

While cleavage-based mechanisms repress protein expression by inducing degradation of mRNA transcripts, ASOs may also be designed to sterically block the assembly and binding of translation initiation machinery, thereby reducing protein expression without directly impacting RNA expression (Figure 2E). This approach may be useful when complete suppression of gene expression is desired, rather than precise modulation of the constituent exons or potential transcripts. Indeed, translation blocking ASOs has been investigated in the context of antiviral development, such as against protein production of Ebola virus,^101^^,^^102^ Marburg virus,^101^ picornaviruses,^103^ and influenza^104^ virus. Translation blocking ASOs are also under investigation as antitumor therapeutics to reduce telomerase activity by targeting translation initiation of the telomerase reverse transcriptase (*TERT*) gene.^105^ Furthermore, a PS-ASO known as oblimersen, which anneals to the AUG initiation site of *BCL2* mRNA, has been evaluated in multiple phase I/II/III trials for various cancers. However, oblimersen is not strictly a translation blocking ASO. While the canonical AUG start codon was identified as an amenable target site, oblimersen is a gapmer and therefore, mediates RNase H1 degradation of the transcript, making it difficult to discern any translation blocking effects. Despite clinical trial involvement, no translation blocking ASOs have received approval, and cleavage-based mechanisms remain the most common to achieve expression knockdown for therapeutic applications. This is perhaps due to sequence limitations, as ASOs must anneal to sequences spanning or adjacent to the canonical AUG initiation codon,^106^^,^^107^^,^^108^ as well as reports of negative feedback loops in which RNA expression of the target gene increases to compensate for the reduction in protein synthesis; although, the effect of this is unknown.^105^

#### Upstream open reading frames

The 5′ end of mRNA transcripts contains several regulatory elements that are crucial for stringent control of gene and protein expression. In particular, the 5′ UTR is the sequence directly upstream of the canonical or primary start codon (pAUG) for the primary open reading frame (pORF) of the mRNApORF, and it is involved in the tight regulation of gene and protein expression.^109^ Typically upwards of 100 nucleotides in length, the 5′ UTR contains various regulatory motifs and elements including internal ribosome entry sites, RNA-binding protein motifs, and TIEs, including secondary structures and upstream open reading frames (uORFs). Recent pre-clinical studies have shown that blocking these regions with ASOs could be a potential mechanism to alter expression of genes that are not amenable to other approaches, such as heterozygous loss-of-function variants leading to protein haploinsufficency. In particular, several studies report the ability to increase protein expression by suppressing uORFs, which may act as potent inhibitors of translation (Figure 2F).^110^^,^^111^^,^^112^^,^^113^ The exact mechanism behind this link remains unknown; however, one theory suggests the increased efficiency of ribosomal scanning.^114^

uORFs consist of an initiation codon upstream of the pAUG and an in-frame termination codon located upstream or within the pORF. Steric blockade of uORFs by ASOs in human and murine genes was first reported in 2017 by Liang et al., in which an 80% increase in murine Lrpprc protein expression was observed following systemic administration of an ASO targeting the uORFs in *Lrpprc* RNA.^110^ This study also reported increased RNase H1 protein expression *in vitro* following treatment with similar uORF-ASOs. However, a 2025 study by Ahlskog et al. could not reproduce this upregulation.^115^ Despite studying the top 3 ASOs reported to increase RNase H1 expression by Liang et al., comprising the same sequence chemical modifications, and analyzing the observations across a wider range of dose and timepoints, Ahlskog et al.^115^ reported the exact opposite effect of RNase H1 translation repression. Diverging observations between the studies may partly reflect differences in methodology, including alternative ASO manufacturers, or the use of an in-house antibody for RNase H1 detection by Liang et al., which could influence isoform specificity and therefore, quantification.

Discrepancies may also arise due to the dynamic nature of the 5′ UTR within the ribosome scanning model. Liang et al. proposed that steric masking of a uORF start codon by an ASO reduces initiation at that site and promotes continued scanning of the complex to the pAUG, where translation is then more efficient in the absence of “false starts” that promote dissociation from mRNA.^116^ However, it is possible that highly stable ASO-mRNA duplexes impede scanning as the complex cannot displace the ASO, causing the complex to dissociate from the mRNA and thereby suppressing translation from the downstream pAUG. This is complicated by the highly context-dependent nature of the 5′ UTR in which the strength of TIE inhibition, and therefore, plausibly uORF suppression, varies between cell types, stage, and stress.^117^ This may underlie the contradictory reports of RNase H1 upregulation by uORF-ASOs, and it may drive the unintended translation repression also reported upon the targeting of uAUG in the 5′ UTR of *GATA4*.^118^ Therefore, a balance between masking uORF suppression while still allowing continued ribosomal scanning may be the key to utilizing this ASO approach.

Beyond limited reproducibility, it is difficult to conclude whether the uORF itself is involved in this effect at all. Indeed, recent studies reported more robust increases in protein expression when ASOs did not overlap the uORF as opposed to those that did,^111^^,^^113^ and some only achieved increased protein expression when treated with both uORF and secondary structure-targeting ASOs in tandem.^112^ Therefore, it is difficult to discern whether interference of the uORF underlies this increase in protein expression or other regulatory elements, such as secondary structures, internal ribosome entry sites, or RNA-binding protein motifs, are responsible. Together with conflicting reports of uORF vs. non-uORF targeting and unintended translation repression, further evidence is required to ascertain the full viability of uORF-ASOs. As such, uORF-ASOs remain in pre-clinical studies.

#### Secondary structures

Another regulatory mechanism within the 5′ UTR that ASOs may be designed to modulate is the tendency of RNA to form strong hybridization strength TIEs that inhibit binding or scanning of translation initiation factors (Figure 2G).^119^ TIEs typically take the shape of G-quadruplex structures, hairpins, or stem-loop structures, and they represent a common naturally occurring regulatory mechanism that ensures tight control of gene expression and translation, such as the iron-responsive element that alters the expression of ferritin in response to iron levels.^120^

Therefore, ASOs that bind within the 5′ UTR and prevent the formation of secondary structures by annealing to sequences involved in their formation are a potential avenue of therapeutic development. Indeed, some studies have reported the potential to increase target protein expression through this mechanism.^112^^,^^118^^,^^121^ A study published in 2017 by Liang et al. reported the upregulation of several human and murine genes by targeting TIEs and secondary structures.^121^ This was validated *in vivo* by increased expression of murine Acp1 after treatment with ASOs targeting the 5′ secondary structure of *Acp1* mRNA. Other studies have reported increased protein expression by targeting 5′UTRs but not specifically uORFs or secondary structures. For example, a 2021 report investigating simultaneous treatment of 5′ and 3′ UTR-targeting ASOs resulted in stable expression of frataxin (*FXN*) mRNA, the causative gene of Friedreich’s ataxia, and increased protein production.^122^ In this case, the ASOs led to increased half-life of the mRNA, and it was concluded that this enhanced stability facilitated increased translation, as there were no reported changes in chromatin-status. However, it is possible this effect could be partly due to modulating other translation inhibitory sites, such as miRNA sites within the 3′ UTR. Similarly, enhanced *SMN2* mRNA stability has also been reported to underlie increased SMN protein expression in SMA patient-derived fibroblasts treated with 5′ UTR targeting ASOs.^123^

Together with targeting uORFs, these studies demonstrate the ability for ASOs to modulate expression of proteins from the canonical reading frame by targeting elements within the 5′ UTRs. However, factors such as the contradictory direction in protein expression changes, questionable reproducibility, and lack of understanding regarding the underlying mechanism of 5′ UTR regulation mean that ASOs operating by this mechanism remain far from the clinic. Nevertheless, this approach could prove promising in disease contexts such as monogenic haploinsufficiency conditions, in which heterozygous inactivating variants in one allele render the remaining allele unable to compensate for required protein levels.

## Conclusion

ASOs represent a uniquely versatile and rapidly evolving class of therapeutics distinguished by their high sequence specificity and continually expanding mechanisms of action. The field has progressed well beyond their early use as simple inhibitors of gene expression to a broad range of strategies capable of modulating RNA biology across multiple stages of gene regulation. Among these, gapmer ASOs are emerging as a promising approach for targeting specific alleles in the context of heterozygous pathogenic variants and for modulating non-coding RNAs to restore or increase expressions of protein-coding genes. While alternative cleavage-mediated mechanisms have been described, their broader therapeutic application remains limited by factors such as stringent sequence requirements and the potential for off-target effects.

In parallel, the repertoire of steric-blocking ASO mechanisms continues to expand. Splice-modulating approaches have evolved beyond restoration of reading frames to include correction of aberrant splicing, modulation of productive and non-productive isoforms, and intentional induction of transcript degradation through reading frame disruption. Additional regulatory layers may also be targeted, including polyadenylation, where modulation of alternative or cryptic polyadenylation sites may be used to alter isoform expression. At the post-transcriptional level, ASOs can modulate miRNA activity through sequestration or by blocking miRNA binding sites on target mRNA. At the level of translation, ASOs may influence both initiation and termination, such as by blocking access to canonical start codons, modulating uORFs or RNA secondary structures, or promoting readthrough of PTCs to restore protein expression. Collectively, these advances highlight the expanding capacity of ASOs to modulate gene expression with increasing precision and flexibility.

Importantly, ASO mechanism of action is influenced by rational design parameters, providing opportunities to bias activity toward specific functional outcomes. Features such as gap length, gap positioning, and chemical modifications can shift ASO behavior toward either RNase H1-mediated cleavage or steric-blocking mechanisms. For example, gap design has been explored to enhance allele selectivity in gapmer approaches, while fully modified backbones and constrained chemistries generally favor RNase H1 resistance and support steric-blocking activity. Despite these advances, systematic rules governing how design parameters dictate mechanism remain undefined.

Despite this mechanistic and design versatility, many emerging ASO strategies remain at an early stage of development. While established mechanisms such as RNase H1-mediated cleavage and splice modulation demonstrate relatively consistent activity across experimental systems, the translational readiness of newer approaches is less defined. In particular, ASO targeting of other RNA regulatory processes remains variable across species as does the translation from cellular models to *in vivo*, potentially limiting translation from pre-clinical models to humans. Although these models provide essential proof-of-mechanism, differences in sequence context, isoform expression, RNA processing, pharmacokinetics, and tissue-specific delivery can significantly influence translational outcomes. Beyond mechanistic feasibility, successful clinical translation requires optimization of delivery, tissue distribution, and pharmacokinetic properties, as well as careful consideration of safety. Specifically, off-target effects remain a critical challenge, arising through both sequence-dependent hybridization and chemistry-related interactions. However, the extent to which mechanism of action contributes to these effects remains unclear. A more integrated understanding of how mechanism, chemistry, and target context influence off-target profiles will be important for guiding future ASO design.

Insight into these challenges is further underscored by ASO candidates that have been discontinued during clinical development for a variety of reasons. For example, the gapmer ASO mipomersen, while demonstrating clinical efficacy, was associated with hepatotoxicity and injection site reactions that limited its long-term use, highlighting the impact of chemistry- and accumulation-related toxicities. In other cases, such as those of AVI-6002 and AVI-6003, promising pre-clinical activity did not translate into sufficient clinical efficacy, likely reflecting challenges in achieving adequate potency and delivery *in vivo*. Together, these examples emphasize that successful translation of ASO therapeutics depends not only on precise modulation of RNA targets, but also on favorable pharmacological and safety profiles, and that mechanistic innovation alone is insufficient to ensure clinical success.

Nevertheless, ASOs are now under investigation for a growing number of conditions previously considered beyond their reach, reflecting their capacity to precisely modulate gene expression in ways not achievable by other therapeutic classes. Continued advances in our understanding of RNA biology, coupled with improvements in ASO design and delivery, will be essential to realize the full therapeutic potential of these approaches. Ultimately, by expanding our mechanistic toolbox through which ASOs can modulate gene and protein expression, we broaden the therapeutic landscape and increase the number of patients who may benefit, while recognizing that substantial work remains to translate these innovations into safe and effective clinical treatments.

## Acknowledgments

We extend our profound gratitude to our colleagues and peers for their invaluable feedback during the drafting process. I.T. is supported by an Australian Government Research Training Program (RTP) stipend and the Prestige Byron Kakulas Scholarship awarded by the 10.13039/501100019111Perron Institute for Neurological and Translational Science.

## Author contributions

Conceptualization, I.T. and J.M.C.; data curation, I.T.; writing – original draft, I.T.; construction of figures and tables, I.T.; writing – review and editing, I.T., S.D.W., J.M.C., and M.A.-H.; supervision, S.D.W., J.M.C., and M.A.-H. All authors have read and agreed to the published version of the manuscript.

## Declaration of interests

S.D.W. is a named inventor on multiple patents relating to splice switching antisense oligomers designed to treat a variety of conditions including DMD, inflammatory disorders, and motor neuron disease and, as such, is entitled to milestone and royalty payments.
